# Do patients with type 1 and type 2 diabetes really have an impaired endothelial function? A population-based propensity score matching analysis

**DOI:** 10.1186/1475-2840-12-174

**Published:** 2013-12-05

**Authors:** Klaus Empen, Roberto Lorbeer, Henry Völzke, Thorsten Reffelmann, Sabine Schipf, Matthias Nauck, Wolfgang Kerner, Henri Wallaschofski, Stephan B Felix, Marcus Dörr

**Affiliations:** 1 Department of Internal Medicine, Medizinische Klinik B, Universitätsmedizin, Greifswald, Sauerbruchstrasse, D-17475, Germany; 2 Institute for Community Medicine, University Medicine, Greifswald 17475, Germany; 3 Institute for Clinical Chemistry and Laboratory Medicine, University Medicine, Greifswald 17475, Germany; 4 Diabetes Center, Karlsburg 17495, Germany; 5 DZHK (German Center for Cardiovascular Research), partner site Greifswald, Greifswald 17475, Germany

**Keywords:** Type 1 diabetes, Type 2 diabetes, Flow-mediated dilation, FMD, Endothelial function, Epidemiology

## Abstract

**Background:**

Previous studies suggested an impaired endothelial function in patients with diabetes. However, the validity of this finding may be limited by the lack of adequate adjustment for further cardiovascular confounders. We assessed endothelial function as measured by flow-mediated dilation (FMD) of the brachial artery in patients with either type 1 or type 2 diabetes in comparison with non-diabetic controls.

**Methods:**

The study population comprised 1487 subjects including 122 subjects with type 2 diabetes, aged 25 to 85, from the population-based Study of Health in Pomerania, and 65 outpatients, aged 23 to 75, with type 1 diabetes. FMD measurements were performed using standardized ultrasound techniques. Subjects with type 1 and type 2 diabetes were matched 1:4 to healthy controls using propensity score matching.

**Results:**

Neither type 1 diabetes (β = 0.142; SE = 0.568, p = 0.803) nor type 2 diabetes (β = 0.107; SE = 0.340, p = 0.752) were significantly associated with FMD in comparison with their non-diabetic controls after adjustment for major cardiovascular confounders like age, gender, body mass index, smoking status, hypertension, antihypertensive medication, LDL and HDL cholesterol levels.

**Conclusions:**

In this population-based study comparing adjusted FMD values of diabetic individuals with adequately matched controls, propensity score analyses revealed no association between diabetes and endothelial function. Since these findings are in discordance with the majority of previous reports, we suggest performing similar analyses using data from other population-based studies.

## Background

Today, the most extensively used non-invasive technique for assessment of endothelial function is sonographic determination of flow-mediated dilation (FMD) of the brachial artery [1]. FMD is considered to represent endothelium-*dependent* vasodilation [1]. Low FMD values predict cardiovascular events independently of established atherosclerosis [2]. Cardiovascular risk factors associated with endothelial dysfunction include hypertension [3], hypercholesterolemia [4], and smoking [5]. Furthermore, type 1 [6-11] and type 2 diabetes [12-17] were reported to be associated with endothelial dysfunction.

Previous studies suggesting an impaired endothelial function in patients with diabetes suffered partly from several limitations such as small sample sizes, different methods of FMD measurements or most importantly lack of adequate selection of control subjects [6-16] as well as non-adjustment for relevant cardiovascular confounders [18]. To overcome these limitations we performed the present propensity score matching analysis for comparison of FMD values of diabetic and non-diabetic subjects using population-based data of the Study of Health in Pomerania (SHIP).

## Methods

SHIP was approved by the ethics committee of the University of Greifswald, and all participants gave informed written consent. Details on the design of SHIP have been published in detail previously [19].

### Controls

Between March 2003 and October 2006, 1788 subjects volunteered for measurement of the FMD of the brachial artery. Exclusion criteria for the measurements were hypotension with systolic blood pressure < 100 mm Hg (*n* = 15), the presence of any other medical contraindication (*n* = 4), and equipment malfunction (*n* = 36). The image quality of 215 FMD examinations was insufficient for appropriate readings. Exclusion of 122 subjects with a history of diabetes mellitus (type 2) according to physician’s diagnosis or use of Antidiabetic medication as well as 31 subjects with hemoglobin A1c levels higher than the diagnostic cut off for diabetes of ≥ 6.5% resulted in a final non-diabetic control population of 1365 subjects (686 women).

### Cases

The first case group consisted of 142 patients with type 1 diabetes recruited from a tertiary Diabetes Care Center in Karlsburg, West Pomerania. Data collection was performed between November 2005 and January 2007. Of these 142 patients, 68 subjects volunteered for measurement of the FMD of the brachial artery. Cases with insufficient image quality of FMD examinations (n = 3) were excluded. Consequently, this case group comprised 65 patients with type 1 diabetes (34 women). The second case group consisted of 122 subjects (54 women) with type 2 diabetes identified in the population-based SHIP.

### Measurements

Information on socio-demographic characteristics, health behaviour, medication intake, and medical history was collected by computer-assisted personal interviews. In the present study we included age, gender, smoking and use of self-reported antihypertensive medication.

Somatometric measurements such as waist circumference, body mass index (BMI), blood pressure were measured as described in detail previously [19,20]. Hypertension was defined as a systolic and/or diastolic blood pressure of ≥140 mmHg and ≥90 mmHg, respectively, or use of antihypertensive medication.

### Assays

Non-fasting blood samples were drawn from the cubital vein in the supine position. The samples were taken between 07.00 a.m. and 04.00 p.m. and analyzed immediately for all parameters. Levels of low and high-density lipoprotein cholesterol (LDL-C and HDL-C), triglycerides and glucose, and HbA1c were determined according to the manufacturer’s recommendations using standard assays as described in detail elsewhere [20]. Internal quality controls were performed at least daily.

### Endothelial function

FMD was assessed as described in more detail previously [20-22] using standardized techniques. All measurements of brachial artery diameters were performed offline by independent sonographers and were subject to strict quality management [22]. In brief, FMD of the brachial artery was assessed by measuring the increase of the brachial artery diameter during reactive hyperemia after transient forearm ischemia induced by interrupting the blood flow for exactly 5 minutes via inflation of a blood pressure cuff placed around the forearm. Post-ischemia diameters were measured one minute after cuff release and restoration of forearm blood flow. End-diastolic vessel diameters were measured from the anterior to the posterior M-line (i.e., the interface between the media and adventitia) of the vessel wall. Absolute FMD were calculated by subtracting baseline vessel diameters from post-ischemia vessel diameters. Relative changes were expressed as percentage of absolute FMD to baseline diameters.

### Statistical analyses

Data on quantitative characteristics are expressed as median with minimum and maximum values. Data on qualitative characteristics are expressed as absolute numbers and percent values. Comparisons between diabetic and non-diabetic population were made using the Mann–Whitney U-test (continuous data) and χ^2^-test (nominal data). Multivariable linear regression models were performed calculating β-coefficients and standard error (SE) for change in FMD between diabetic (type 1 and 2) and non-diabetic subjects. The models were adjusted for age, gender, smoking, BMI, hypertension, antihypertensive medication, LDL cholesterol (LDL-C) and HDL cholesterol (HDL-C) levels.

We used propensity score matching to estimate FMD in subjects with similar characteristics of diabetic and non-diabetic groups. The likelihood of having type 1 or type 2 diabetes mellitus was modelled in a logistic regression depending on age, gender, BMI, current smoking, hypertension, LDL-C and HDL-C levels and the predicted probability was computed for each subject [23]. For propensity score matching we used the nearest neighbour matching method. In the event of ties, a control was selected at random from among all potential matches.

For the power calculation we considered different ratios for propensity score matching (1:2, 1:3 or 1:4). The ratios were varied until a meaningful difference in FMD means of 1.5% was detectable with a power of 80% at least (alpha_2-sided_, 0.05; common standard deviation, 3.8;). The ratio was increased until the power calculation resulted in a number of subjects with type 1 diabetes mellitus ≤ 65. We chose a 1:4- matching where the power calculation resulted in a number of necessary subjects of 63, which was lower than the number patients with type 1 diabetes (n = 65). The final samples for the matched comparisons comprised 276 subjects for type 1 diabetes analyses (65 diabetic and 211 non-diabetic; power: 79.5%) and 430 subjects for type 2 diabetes analyses (120 diabetic and 310 non-diabetic; power: 95.7%) without missing data for confounder variables.

Bivariate comparisons were performed on the unmatched groups and multivariable regression analyses were performed on the matched groups. A value of p < 0.05 was considered statistically significant. All statistical analyses were performed using Stata 12.1 (Stata Corporation, College Station, TX, USA).

## Results

Baseline characteristics of the non-diabetic and the diabetic groups are presented in Table 1. Subjects with type 1 diabetes showed a higher proportion of hypertension, increased HDL-C levels and decreased LDL-C levels as well as lower baseline diameter of the brachial artery compared to the non-diabetic group. There were no significant differences between these two groups concerning age, smoking status, BMI, systolic blood pressure, and FMD (Table 1). Subjects with type 2 diabetes were different from non-diabetic subjects in all analyzed parameters except the gender proportion.

**Table 1:** Characteristics of subjects with and without diabetes

	**SHIP-population**		**Outpatients**
	**Non diabetic**	**Type 2 diabetes**	**Type 1 diabetes**
	**n = 1365**	**n** = **122**	**n =65**
Age, yr	51 (25–85)	63 (31–84)^‡^	50 (23–75)
Sex (male)	679 (49.7%)	68 (55.7%)	31 (47.7%)
Smoking status		^‡^	
Never-smoker	590 (43.3%)	48 (39.3%)	32 (49.2%)
Ex-smoker	427 (31.3%)	61 (50.0%)	14 (21.5%)
Current smoker	347 (25.4%)	13 (10.7%)	19 (29.2%)
Body mass index, kg/m^2^	27.0 (16.8–49.8)	31.0 (19.8–50.0)^‡^	26.5 (20.0–41.5)
Waist circumference, cm	91.3 (57.3–155.0)	103.0 (75.2–135.0)^‡^	91.2 (63.0–126.1)
Systolic blood pressure, mmHg	129 (89–219)	135 (90–208)^‡^	137 (101–179)
Diastolic blood pressure, mmHg	82 (54–122)	79 (57–113)^†^	78 (47–101)*
Hypertension	623 (45.7%)	92 (75.4%)^‡^	41 (63.1)^†^
Use of antihypertensive medication	418 (30.6%)	98 (80.3%)^‡^	37 (56.9%)^‡^
HDL cholesterol, mmol/l	1.13 (0.39–2.82)	0.87 (0.42–2.22)^‡^	1.35 (0.53–2.84)^‡^
LDL cholesterol, mmol/l	3.46 (0.73–11.03)	3.19 (1.45–5.99)*	2.98 (1.64–5.06)^†^
Triglycerides, mmol/l	1.43 (0.17–52.22)	1.99 (0.32–13.27)^‡^	1.04 (0.31–4.74)^‡^
Use of sex hormones medication	126 (9.2%)	1 (0.8%)^†^	2 (3.1%)
Glucose, mmol/l	5.1 (3.3–11.1)	6.4 (2.4–20.2)^‡^	8.4 (1.5–22.8)^‡^
HbA1c,%	5.2 (2.8–6.4)	6.5 (3.7–10.1)^‡^	7.5 (4.3–11.7)^‡^
Albumin, mg/l	5.4 (0–2120)	10.6 (0–2880)^‡^	12.6 (2.4–756)^‡^
Baseline diameter A.brachialis, mm	3.84 (2.10–6.01)	4.07 (2.21–6.01)^‡^	3.78 (2.54–4.99)*
FMD,%	4.51 (-8.44–29.41)	3.89 (-3.50–11.13)*	4.21 (-1.32–14.79)

Data are given as number (percentage) or median (minimum-maximum).

*p < 0.05, ^†^p < 0.01, ^‡^p < 0.001 c2-test (nominal data) or Mann-Whitney U test (continuous data).

Comparisons were performed separately against non-diabetics.

HDL, high-density-lipoprotein; LDL, low-density-lipoprotein; HbA1c, hemoglobin A1c; FMD, flow-mediated dilatation.

Linear regression analyses revealed no difference in adjusted FMD between patients with type 1 diabetes and non-diabetic subjects (β = 0.142; SE = 0.568, p = 0.803) (Table 2). Likewise, there was no difference in adjusted FMD between patients with diabetes type 2 and non-diabetic subjects (β = 0.107; SE = 0.340, p = 0.752) (Table 3).

**Table 2:** Multivariable linear regression analysis in volunteers with type 1 diabetes and non-diabetic matched controls with respect to FMD

	**FMD**		
**N = 276**	**β-coefficients**	**SE**	**p-value**
Type 1 diabetes	0.142	0.568	0.803
Age, years	-0.067	0.023	0.004
Sex (male)	-1.645	0.534	0.002
Current smoking	-0.099	0.561	0.860
Body mass index, kg/m^2^	0.063	0.061	0.307
Hypertension	-0.444	0.571	0.438
Use of antihypertensive medication	-0.361	0.612	0.556
HDL cholesterol, mmol/l	0.151	0.555	0.786
LDL cholesterol, mmol/l	0.288	0.293	0.327

FMD, flow-mediated dilatation; HDL, high-density-lipoprotein; LDL, low-density-lipoprotein.

**Table 3:** Multivariable linear regression analysis in volunteers with type 2 diabetes and non-diabetic matched controls with respect to FMD

	**FMD**		
**N = 430**	**β-coefficients**	**SE**	**p-value**
Type 2 diabetes	0.107	0.340	0.752
Age, years	-0.072	0.014	<0.001
Sex, male	-1.418	0.328	<0.001
Current smoking	-0.040	0.450	0.930
Body mass index, kg/m^2^	-0.038	0.035	0.282
Hypertension	0.373	0.366	0.308
Use of antihypertensive medication	-0.423	0.374	0.259
HDL cholesterol, mmol/l	-0.118	0.467	0.801
LDL cholesterol, mmol/l	-0.088	0.172	0.609

FMD, flow-mediated dilatation; HDL, high-density-lipoprotein; LDL, low-density-lipoprotein.

In a sensitivity analysis, there was no association between HbA1c levels and FMD neither in type 1 diabetes (β = -0.099; SE = 0.437, p = 0.821) nor in type 2 diabetes (β = 0.266; SE = 0.207, p = 0.201) adjusted for age, gender, smoking, BMI, hypertension, antihypertensive medication, LDL and HDL cholesterol levels. To minimize potential misclassification bias concerning undiagnosed diabetes in control subjects, we repeated propensity score matching with controls with an HbA1c level < 5.7%. This analysis revealed similar results showing also no associations between diabetes and FMD neither for type 1 (β = 0.274; SE = 0.538, p = 0.611) nor for type 2 diabetes (β = 0.208; SE = 0.338, p = 0.539).

## Discussion

In contrast to previous studies we did not find an association of either type 1 [6-11] or type 2 [12-16] diabetes with impaired endothelial function. Our deviating findings may be explained by a more sophisticated selection of controls by using propensity score matching and considering known confounders, i.e. gender, age, body mass index, smoking, hypertension, LDL-C and HDL-C levels, as well as use of antihypertensive medication.

Selection of healthy controls remains an issue of concern of many previous preclinical and clinical studies [24]. Controls in studies investigating the association between diabetes and endothelial function were often recruited from convenience samples like members of hospital staff, their relatives, or friends (e.g. [8]). However, lack of adequate control groups cannot be attributed to every single of previous studies investigating endothelial function in patients with type 1 diabetes. For example, cardiovascular risk factors appeared well-balanced between patients with type 1 diabetes and controls in one of these studies [25]. Interestingly, in this study FMD was lower in diabetic patients, but this association was no longer observed after correction for baseline diameters. However, participants of this study were significantly younger than in SHIP (median age 32 years) and consequently did not present further cardiovascular risk factors with the exception of smoking. According to a meta-analysis [26], the association of FMD with cardiovascular risk is limited to populations at relatively low cardiovascular risk. Due to the prevalence of further cardiovascular risk factors the resulting estimated general cardiovascular risk in our subjects was not as low as in other studies [7,9]. Thus, the relatively high cardiovascular risk of our volunteers might partly explain the lack of association of type 1 diabetes and FMD observed in the present study. In partial concordance with this assumption are the results of a recent study in children with type 1 diabetes with a disease duration > 5 years who did not present with excessive additional cardiovascular risk revealing an impaired FMD in children with diabetes compared to controls [27]. In contrast, another recent study did not observe impaired FMD at baseline in children and adolescents with type 1 diabetes and a mean disease duration of 4 years, but did observe an impairment after a mean follow-up of 3 years [28]. Our volunteers were adults with a mean age of 50 years, mostly long-lasting type 1 diabetes, and a high prevalence of additional cardiovascular risk factors which makes a direct comparison difficult.

With respect to diabetes mellitus type 2 previous studies mostly described an association with decreased endothelial function. For example, the HOORN study with a larger sample size than ours described lower FMD values in type 2 diabetics compared to controls with normal glucose metabolism [13]. This association was still present after adjustment for conventional cardiovascular risk factors, which were of similar prevalence as in our group of subjects with type 2 diabetes. In this study, however, impaired glucose metabolism was not associated with impaired FMD. In a more recent study of 165 patients with diabetes mellitus type 2, FMD was inversely associated with duration of diabetes, but there was no association of FMD with other cardiovascular risk factors [17]. Unfortunately, we are unable to confirm this finding because precise data on disease duration are not available in a relevant proportion of our volunteers with type 2 diabetes.

Contradictory findings concerning the association of type 2 diabetes and FMD may be potentially partly explained by inconsistent definitions of type 2 diabetes. In our study, the definition was based on self-reported physician’s diagnosis or use of antidiabetic medication. Although volunteers with an HbA1c value greater than 6.5% were excluded from the control group, because they can be considered diabetic according to current diagnostic guidelines [29], we cannot completely exclude that an uncertain proportion of control volunteers had undiagnosed type 2 diabetes leading to a misclassification bias. However, the probability of including a relevant proportion of subjects with undiagnosed type 2 diabetes in the controls appears low since reanalysis of the data after including only subjects with HbA1c levels lower than 5.7% as controls did not reveal different results.

In our sensitivity analysis, there were no associations of HbA1c levels with FMD, neither in type 1 nor in type 2 diabetes. In a previous study in a non-diabetic population, we observed an inverse association of HbA1c only in women, but not in men [20]. In the current study, we considered numbers of subjects with diabetes too low for performing gender-specific analyses. Most previous authors reported impaired endothelial function in type 1 and 2 diabetes, but not all investigators did confirm this association [25]. Considering the effects of publication bias, the association may be truly neutral – as suggested by our data analysis.

Major strengths of our study are the population-based design, the comprehensive and detailed assessment of potential metabolic and cardiovascular confounding factors [22], the size of the study population, the accurate FMD measurement under strict quality management by standardised protocol and certified staff [22], and the use of forearm ischemia-induced FMD [1]. Forearm ischemia-induced FMD precludes the potential contribution of ischemia of the brachial artery itself – in contrast to upper arm ischemia-induced FMD which is known to induce greater vasodilation [1]. Finally, and probably most importantly, our analysis is the first one selecting controls out of the same population by propensity score matching.

## Conclusion

In the present analysis, there was no association of either type 1 or type 2 diabetes with impaired endothelial function as measured by FMD. Since these findings are in discordance with the majority of previous reports, we suggest performing similar analyses using data from other population-based studies.

## Abbreviations

BMI: Body mass index; FMD: Flow-mediated dilation; HDL-C: High density lipoprotein cholesterol; LDL-C: Low density lipoprotein cholesterol; SE: Standard error; SHIP: Study of Health in Pomerania.

## Competing interests

The authors declare that they have no competing interests.

## Authors’ contributions

KE, RL, MD designed the study, researched data, and wrote the first draft of the manuscript. RL performed statistical analyses. HV, TR, SS, MN, HW, SBF researched data, contributed to discussion, and reviewed the manuscript. WK recruited patients with type 1 diabetes and reviewed the manuscript. All authors have approved the final version of the manuscript.
